# Treatment of Ileus

**Published:** 1892-02

**Authors:** 


### THE TREATMENT OF ILEUS.

Dr. Aufrecht attributes the sudden arrest of vomiting when the stomach is distended to a dislocation which is produced by the distended intestine forcing the œsophagus against the edge of the opening in the diaphragm through which the œsophagus passes. In stercoracecus vomiting, repeated washings of the stomach will not only act beneficially by removing the contents, but will prevent injury from absorption. Morphine, hypodermatically, is advised in all cases of ileus, and large enemata have been abandoned.—*Therapiutische Monatshefte.*

